# Attitudes to routine HIV counselling and testing, and knowledge about prevention of mother to child transmission of HIV in eastern Uganda: a cross-sectional survey among antenatal attendees

**DOI:** 10.1186/1758-2652-13-52

**Published:** 2010-12-13

**Authors:** Robert Byamugisha, James K Tumwine, Grace Ndeezi, Charles AS Karamagi, Thorkild Tylleskär

**Affiliations:** 1 Department of Obstetrics and Gynaecology, Mbale Regional Referral Hospital, PO Box 921, Mbale, Uganda; 2 Department of Paediatrics and Child Health, School of Medicine, Makerere University College of Health Sciences, PO Box 7072, Kampala, Uganda; 3 Centre for International Health, University of Bergen, Postbox 7804, N-5020 Bergen, Norway

## Abstract

**Background:**

HIV testing rates have exceeded 90% among the pregnant women at Mbale Regional Referral Hospital in Mbale District, eastern Uganda, since the introduction of routine antenatal counselling and testing for HIV in June 2006. However, no documented information was available about opinions of pregnant women in eastern Uganda about this HIV testing approach. We therefore conducted a study to assess attitudes of antenatal attendees towards routine HIV counselling and testing at Mbale Hospital. We also assessed their knowledge about mother to child transmission of HIV and infant feeding options for HIV-infected mothers.

**Methods:**

The study was a cross-sectional survey of 388 women, who were attending the antenatal clinic for the first time with their current pregnancy at Mbale Regional Referral Hospital from August to October 2009. Data were collected using a pre-tested questionnaire and analysed using descriptive statistics and logistic regression. Permission to conduct the study was obtained from the Makerere University College of Health Sciences, the Uganda National Council of Science and Technology, and Mbale Hospital.

**Results:**

The majority of the antenatal attendees (98.5%, 382/388) had positive attitudes towards routine HIV counselling and testing, and many of them (more than 60%) had correct knowledge of how mother to child transmission of HIV could occur during pregnancy, labour and through breastfeeding, and ways of preventing it. After adjusting for independent variables, having completed secondary school (odds ratio: 2.5, 95% confidence interval: 1.3-4.9), having three or more pregnancies (OR: 2.5, 95% CI: 1.4-4.5) and belonging to a non-Bagisu ethnic group (OR: 1.7, 95% CI: 1.0-2.7) were associated with more knowledge of exclusive breastfeeding as one of the measures for prevention of mother to child transmission of HIV. Out of 388 antenatal attendees, 386 (99.5%) tested for HIV and 382 (98.5%) received same-day HIV test results.

**Conclusions:**

Routine offer of antenatal HIV counselling and testing is largely acceptable to the pregnant women in eastern Uganda and has enabled most of them to know their HIV status as part of the prevention of mother to child transmission of HIV package of services. Our findings call for further strengthening and scaling up of this HIV testing approach in many more antenatal clinics countrywide in order to maximize its potential benefits to the population.

## Background

HIV counselling and testing is pivotal to HIV prevention, care and treatment programmes as knowing one's HIV status is a precursor to accessing the appropriate care and treatment services. However, data from surveys conducted in 12 high-prevalence countries in sub-Saharan Africa show that only 12% of men and 10% of women know their HIV status [1]. Uganda has an estimated adult HIV prevalence rate of 6.7%, and only 15% of adults are aware of their HIV status [2]. It is estimated that in 2008, mother to child transmission of HIV accounted for 15% of new HIV infections in Uganda [3,4].

Routine antenatal counselling and testing for HIV, also known as provider-initiated testing or an "opt-out" approach, involves testing all antenatal attendees for HIV, apart from those who decline the test (i.e., those who opt out). This is the standard of care in Scandinavia and other high-income countries [5-10]. In a bid to increase HIV testing rates, routine antenatal HIV counselling and testing was successfully introduced in the HIV prevention programmes of several countries in sub-Saharan countries [11-15] in line with Centers for Disease Control and Prevention (CDC) and Joint United Nations Programme on HIV/AIDS (UNAIDS) and World Health Organization (WHO) recommendations [16,17].

In Uganda, the policy change from antenatal voluntary HIV counselling and testing (VCT), also known as the client-initiated or "opt-in" approach (where clients are encouraged to undergo counselling and testing for HIV if they so wish) to routine HIV counselling and testing (RCT) was integrated into the prevention of mother to child transmission (PMTCT) of HIV programme in 2006 [18]. As a result, there has been a sustained increase in HIV testing rates to more than 90% among the pregnant women at Mbale Regional Referral Hospital since June 2006 [19]. We conducted our study to assess: (a) attitudes towards routine HIV testing among the new antenatal attendees in the hospital; and (b) their knowledge about mother to child transmission of HIV and infant feeding options for HIV-infected mothers.

## Methods

The study site was Mbale Regional Referral Hospital, located in the town of Mbale, approximately 240 kilometres north-east of the city of Kampala by road, in Mbale District. The district has a population of about 410,600 (2010 estimate) [20] and an annual population growth rate of 2.5%, according to the 2002 national census. The majority (92%) of the people in this district live in the rural areas. They are predominantly Bagisu or Bamasaba people. The main language is Lumasaba and the main economic activity is subsistence farming. The literacy rate is 64% for men and 49% for women [21]. In 2003, HIV prevalence was reported to be 5.6% [22].

The hospital in Mbale is a regional referral hospital for 11 districts in eastern Uganda and serves an estimated population of 1.9 million people. The hospital has a bed capacity of 380 and serves 6000 to 9000 new antenatal attendees per year. Antenatal care services (ANC) are provided daily, except for weekends. The average attendance is 50 to 60 pregnant women per day, including those who come for ANC return visits.

The prevention of mother to child transmission (PMTCT) of HIV programme was launched at the hospital in May 2002 as an integrated service in the antenatal care services. The PMTCT programme was introduced as a voluntary counselling and testing (VCT) for HIV approach. In line with CDC and UNAIDS/WHO recommendations [16,17], the Uganda Ministry of Health issued new guidelines for HIV counselling and testing of pregnant women in September 2005 [23] and a revised edition of Policy Guidelines for Prevention of Mother to Child Transmission of HIV in August 2006 [18]. VCT was replaced by routine counselling and testing (RCT) in Mbale Regional Referral Hospital in June 2006.

Currently, service providers give pre-test group counselling to groups of ANC attendees. The counsellors in the antenatal clinic first attend to couples who come for RCT when the clinic opens in the morning before attending to the mothers who have come alone: this is one way of encouraging couple attendance. Routine HIV testing is done with the client's knowledge and verbal consent. Mothers are free to decline (opt out of) HIV testing if they so wish without fear of any retribution from the clinic staff.

According to national guidelines, a sequential HIV testing algorithm, with same-day results, including three rapid tests is used on one blood sample: Determine HIV 1 ⁄ 2 assay (Abbott Laboratories, Abbott Park, IL, USA) for first screening; STAT-PAK HIV 1 ⁄ 2 dipstick assay (Chembio Diagnostic Systems Inc.) as a second test and Uni-Gold Recombigen HIV (Trinity Biotech, Wicklow, Ireland) as a "tie-breaker". An ANC attendee is classified as uninfected if Determine is negative and as HIV-infected if both Determine and STAT-PAK tests are positive. Discordant Determine and STAT-PAK blood samples are tested using the Uni-Gold test. The HIV test result is reported as positive if the Uni-Gold test is positive, or as negative if both STAT-PAK and Uni-Gold tests are negative. Since 2006, ANC attendees who test HIV positive undergo CD4 cell count tests before being given appropriate treatment according to the national PMTCT guidelines [18].

A cross-sectional survey was conducted among 388 new antenatal attendees in the antenatal clinic at Mbale Regional Referral Hospital from August to October 2009. The targeted study population were all antenatal attendees who were visiting the hospital for the first time within the current pregnancy. Women, who were very sick, requiring urgent medical attention, were excluded from the study. Women attending ANC for the first time were identified at reception, and tracked through RCT for HIV and through routine antenatal assessment. All those who were confirmed as having undergone RCT for HIV were consecutively identified and approached for inclusion in the study after giving written informed consent for exit interviews about their attitudes regarding RCT until the required sample size was obtained.

The sample size was calculated using the computer programme, OpenEpi, version 2, open source calculator (open source software for epidemiologic statistics: http://www.openepi.com/SampleSize/SSCohort.htm), based on the following assumptions: (a) a two-sided confidence level or interval of 95% (level of significance of 5%); and (b) a 50% prevalence of positive attitudes about RCT among the antenatal attendees.

A standardized, pre-tested questionnaire was administered in either English or Lumasaba by five trained research assistants. The questionnaire was adapted from a pilot project on routine HIV testing in Botswana [14] and had 50 items. The structured interview covered topics concerning the participant's and her partner's education, occupation, religion, ethnic group, number of pregnancies, household assets, opinions and experiences about routine HIV counselling and HIV testing in the antenatal clinic, and knowledge about mother to child transmission of HIV and infant feeding options for HIV-infected mothers. Exclusive breastfeeding (EBF) was defined in this study as feeding an infant with only breast milk and nothing else, even water, apart from prescribed medicines or vitamins. During group counselling sessions in the antenatal clinic, counsellors discussed the lactational amenorrhea that occurs as a result of EBF.

The research assistants were knowledgeable in the local language and interview techniques, and had received training about the study objectives and methods. The principal investigator checked filled questionnaires for completeness at the end of each day. Data-entry clerks entered data, using EpiData version 3.1; the principal investigator undertook validation of data, checking for any errors in the data in EpiData file. We exported the data file to PASW Statistics 18 (formerly SPSS) for analysis.

Ethical clearance to conduct the study was obtained from the Research and Ethics Committee of the School of Medicine, Makerere University College of Health Sciences, and the Uganda National Council of Science and Technology. Permission to conduct the study in the antenatal clinic was also obtained from the Mbale Regional Referral Hospital administration through the local institutional review board.

The main outcome measure was a positive attitude of pregnant women to routine counselling and testing for HIV. The secondary outcome was participants' knowledge about mother to child transmission of HIV and infant feeding options for HIV-infected mothers. We used descriptive statistics to examine the demographic characteristics of the participants and their experiences with and attitudes towards RCT. The participants were grouped into socio-economic quintiles based on a proxy wealth index using principal component factor analysis [24]. Housing characteristics and assets, including radio, hurricane lamp, television set, mobile phone, bicycle, motorcycle, motor vehicle, refrigerator, sofa and cupboard, were included in the model.

Prior to performing the principal component analysis, the suitability of the data for factor analysis was assessed. The correlation matrix showed some coefficients of 0.3 and above. The Kaiser-Meyer-Oklin of Sampling Adequacy value was 0.808, exceeding the value of ≥ 0.6 recommended for this test to demonstrate that factors are inter-correlated, and the Barlett's test of Sphericity was significant (p = 0.000), supporting the factorability of the correlation matrix [25]. The quintiles were based on the first principal component, a recognized method to provide a good proxy for household wealth [26,27]. Participants were asked, "Nowadays in this clinic, all mothers are tested for HIV unless they say no. What do you think about this system?" Responses included "very bad", "bad", "fair", "good" and "very good". The responses, "good" and "very good", were taken as positive attitudes towards routine HIV testing.

Bivariate analysis was performed between knowledge about exclusive breastfeeding as an infant feeding option by HIV-infected mothers as the dependent variable and each independent (predictor) variable. Bivariate analysis was also performed between each independent variable and the following dependent variables: positive attitude to pre- and post-test HIV counselling and to HIV testing; and having sought male partner permission to test for HIV. Multicollinearity among the independent variables and outliers were checked for.

Age as a possible confounder and all variables that were significant at the level of p < 0.2 in binary analysis were retained in the multivariate regression model. All p-values were two-tailed at a significance level of 5%. The goodness-of-fit test (Omnibus Tests of Model Coefficients) of the final model for knowledge about exclusive breastfeeding was significant [Chi-square statistic (χ^2^) = 28.249, degrees of freedom (df) = 7, p = 0.000] and the Hosmer and Lemeshow goodness-of-fit test was not significant (χ^2 ^= 5.866, df = 8, p = 0.662) as indicators of model appropriateness. The final models for positive attitude to pre-test and post-test counselling, HIV testing and having sought male partner permission for HIV tests yielded Hosmer and Lemeshow goodness-of-fit test results that were not significant (p-value > 0.05).

## Results

### Socio-demographic characteristics

Of the 388 new antenatal attendees enrolled in the study, about two-thirds were living in rural villages, and they had a median age of 24 years (range 15-46 years, Table 1). Most of them were Christians, had no salaried employment, were in a consensual relationship, and had less than 11 years of education (74%). The majority (64%) of the participants were Bagisu, and most of them had had at least one previous pregnancy. Their male partners had a median age of 30 years (range 18-72 years) and about half of them had completed secondary education.

**Table 1:** Predictors of knowledge of exclusive breastfeeding among 388 new antenatal attendees, Mbale, Uganda; logistic regression results

Participants' characteristics	Number, n (%)	Exclusive breastfeeding knowledge	
		
		Unadjusted OR (95% CI)	Adjusted OR (95% CI)
*Age groups (years)*			
15-24	220 (56.7)	1.0	1.0
25 or more	168 (43.3)	1.1 (0.7-1.7)	1.5 (0.8-2.5)
*Place of residence*			
Rural	252 (64.9)	1.0	
Urban	136 (35.1)	1.5 (0.9-2.3)	1.4 (0.9-2.3)
*Marital status*			
Single/divorced/separated	35 (9.0)	1.0	
Married/cohabiting	353 (91.0)	1.4 (0.7-2.8)	
*Occupation*			
Not getting a salary	337 (86.9)	1.0	1.0
Salaried	51 (13.1)	2.6 (1.2-5.6)	1.9 (0.8-4.5)
*Education level*			
None or incomplete primary	134 (34.5)	1.0	1.0
Completed primary	152 (39.2)	1.5 (0.9-2.4)	1.6 (1.0-2.7)
Completed secondary or more	102 (26.3)	2.5 (1.4-4.5)	**2.5 (1.3-4.9)**
*Religion*			
Christian	234 (60.3)	1.0	
Muslim	154 (39.7)	1.2 (0.8-1-9)	
*Number of pregnancies*			
1-2	201 (51.8)	1.0	1.0
3 or more	187 (48.2)	1.5 (1.0-2.3)	**2.5 (1.4-4.5)**
*Socio-economic status*			
Poorest (quintiles: 4^th^, 5^th^)	159 (41.0)	1.0	
Least poor (quintiles: 1^st^-3^rd^)	229 (59)	1.3 (0.8-1.9)	
*Ethnic group*			
Bagisu	247 (63.7)	1.0	1.0
Non-Bagisu	141 (36.3)	1.6 (1.0-2.5)	**1.7 (1.0-2.7)**
*Tested for HIV today *^**a**^			
No	2 (0.5)		
Yes	386 (99.5)		
*Received same-day HIV test results*			
No	6 (1.5)	1.0	
Yes	282 (98.5)	1.9 (0.4-9.8)	

I. ^**a**^No unadjusted odds ratio was calculated since one of the cells had less than 5 cases.

II. P-value (P) < 0.05 was statistically significant.

III. The goodness-of-fit test (Omnibus Tests of Model Coefficients) of the final model was significant [Chi-square statistic (χ^2^) = 28.249, degrees of freedom (df) = 7, p = 0.000] and the Hosmer and Lemeshow goodness-of-fit test was not significant [χ^2 ^= 5.866, df = 8, p = 0.662] as indicators of model appropriateness.

### Overall results

Almost all the new ANC attendees (98.5%, 382/388) had a positive attitude towards routine HIV testing in the clinic. They reported that it helped them to know their HIV status and that this in turn enabled them to plan for their future and that of their babies. They also reported that mothers found to be HIV positive would be able to easily access antiretroviral therapy to reduce the risk of transmitting HIV to their babies. However, mothers with negative HIV test results would protect themselves from getting infected with HIV.

### Participants' opinions and experiences of routine HIV counselling and testing

The majority of the study participants reported that their first visits to the antenatal clinic for the current pregnancy had been good and that they were handled well by the clinic staff (Table 2). Most of the women rated highly the health education talk and the pre-test and post-test HIV counselling they had received at the clinic. The predictors of positive attitude to pre-test counselling for HIV included: residing in an urban area (OR: 3.0, CI: 1.4-6.6); being least poor (OR: 1.9, CI: 1.0-3.7); having three or more pregnancies (OR: 3.0, CI: 1.4-6.8); and being 15 to 24 years of age (OR: 2.5, CI: 1.1-5.4) (Table 3).

**Table 2:** Participants' opinions and experiences about routine HIV testing among 388 new antenatal attendees, Mbale, Uganda

Participant's rating of	Responses	
	
	Very good/good n (%)	Fair/bad/very bad n (%)
the visit to antenatal clinic	308 (79.4)	80 (20.6)
the handling by clinic staff	344 (88.7)	44 (11.3)
the total waiting time in clinic	286 (73.7)^**a**^	102 (26.3)^**b**^
the clinic facilities	322 (83.0)	66 (17.0)
the health education talk	350 (90.2)	38 (9.8)
the pre-test HIV counselling	335 (86.3)	53 (13.7)
the post-test HIV counselling	369 (95.1)	19 (4.9)
the routine HIV testing^**c**^	382 (98.5)	6 (1.5)

^**a **^Not long waiting time

^**b **^Too long waiting time

^**c **^Participants were asked, "Nowadays in this clinic, all mothers are tested for HIV unless they say no. What do you think about this system?" Responses included "very bad", "bad", "fair", "good" and "very good". The responses, "good" and "very good", were taken as positive attitudes towards routine HIV testing.

**Table 3:** Predictors of positive attitude to pre-test HIV counselling among 388 new antenatal attendees, Mbale, Uganda

Participants' characteristics	Number n (%)	Pre-test HIV counselling positive attitude	
		
		Unadjusted OR (95% CI)	Adjusted OR (95% CI)
*Age groups (years)*			
25 or more	168 (43.3)	1.0	1.0
15-24	220 (56.7)	1.2 (0.7-2.1)	**2.5 (1.1-5.4)**^**‡**^
*Place of residence*			
Rural	252 (64.9)	1.0	1.0
Urban	136 (35.1)	3.0 (1.4-6.3)	3.0 (1.4-6.6)^*^
*Education level*			
No or incomplete primary	134 (34.5)	1.0	1.0
Completed primary	152(39.2)	0.9 (0.5-1.9)	0.7 (0.4-1.5)
Completed secondary or more	102 (26.3)	1.8 (0.8-4.2)	1.3 (0.5-3.5)
*Ethnic group*			
Non-Bagisu	141 (36.3)	1.0 (0.6-1.9)	
Bagisu	247 (63.7)	1.0	
*Socio-economic status*			
Poorest (quintiles: 4^th^-5^th^)	159 (41.0)	1.0	1.0
Least poor (quintiles: 1^st^-3^rd^)	229 (59.0)	2.3 (1.3-4.1)	**1.9 (1.0-3.7)**^**‡**^
*Occupation*			
Salaried	51 (13.1)	1.0	1.0
Not getting a salary	337 (86.9)	2.8 (0.8-9.3)	1.8 (0.5-7.0)
*Number of pregnancies*			
1-2	201 (51.8)	1.0	1.0
3 or more	187 (48.2)	1.5 (0.8-2.3)	3.0 (1.4-6.8)^*^

I. P-value: **‡ **= p < 0.05, * = p < 0.01

II. Marital status and religion were not significantly associated with positive attitude to pre- and post-test HIV counselling.

III. The goodness-of-fit test (Omnibus Tests of Model Coefficients) of the final model for pre-test counselling positive attitude was significant [Chi-square statistic (χ^2^) = 17.219, degrees of freedom (df) = 7, p = 0.016] and the Hosmer and Lemeshow goodness-of-fit test was not significant [χ^2 ^= 9.620, df = 8, p = 0.293] as indicators of model appropriateness.

Out of 388 new antenatal attendees, 386 (99.5%) tested for HIV and 382 (98.5%) received same-day HIV test results (Table 1). In addition, 54% (211/388) of the women had sought their partners' permission to test for HIV in the antenatal clinic and almost all of them (209/211, 99%) got this permission. The predictors for male partner permission for the HIV test were: being married or cohabiting (OR: 5.6, CI: 2.4-13.3); and having completed secondary school education or more (OR: 3.0, CI: 1.5-5.9) (Table 4). Nearly all the study participants rated highly the routine HIV testing services offered as part of standard antenatal care (Table 2).

**Table 4:** Predictors of male partner permission to test for HIV and positive attitude to HIV testing among 388 new antenatal attendees, Mbale, Uganda

Participants' characteristics	Number n (%)	Male partner permission to test for HIV		Positive attitude to HIV-testing^†^	
		
		Unadj.OR (95% CI)	Adj.OR (95% CI)	Unadj.OR (95% CI)	Adj.OR (95% CI)
*Age groups (years)*					
15-24	220 (56.7)	1.0	1.0	2.7 (0.5-14.7)	2.5 (0.3-22.3)
25 or more	168 (43.3)	1.2 (0.8-1.8)	1.0 (0.7-1.6)	1.0	1.0
*Education level*					
No or incomplete primary	134 (34.5)	1.0	1.0		
Completed primary	152 (39.2)	1.2 (0.8-1.9)	1.2 (0.7-2.0)		
Completed secondary or more	102 (26.3)	2.7 (1.5-4.7)	3.0 (1.5-5.9)^*^		
*Socio-economic status*					
Poorest (quintiles: 4^th^-5^th^)	159 (41.0)	1.0	1.0	1.0	1.0
Least poor (quintiles: 1^st^-3^rd^)	229 (59.0)	1.5 (1.0-2.2)	1.2 (0.7-1.9)	2.9 (0.5-16.2)	1.9 (0.3-12.6)
*Ethnic group*					
Bagisu	247 (63.7)	1.0	1.0	1.0	1.0
Non-Bagisu	141 (36.3)	1.6 (1.0-2.4)	1.6 (1.0-2.5)	2.9 (0.3-25.0)	2.6 (0.3-23.9)
*Marital status*					
Single/divorced/separated	35 (9.0)	1.0	1.0	1.0	1.0
Married/cohabiting	353 (91.0)	4.6 (2.0-10.5)	**5.6 (2.4-13.3)**^**‡**^	2.0 (0.3-18.0)	5.4 (0.4-73.1)
*Religion*					
Christian	234 (60.3)	1.0	1.0	1.0	1.0
Moslem	154 (39.7)	1.3 (0.9-2.0)	1.4 (0.9-2.2)	1.3 (0.2-7.3)	1.2 (0.2-7.0)
*Occupation*					
Not getting a salary	337 (86.9)	1.0	1.0		
Salaried	51 (13.1)	1.9 (1.0-3.6)	1.1 (0.5-2.3)		
*Education level*					
No or incomplete primary	134 (34.5)			1.0	1.0
Completed primary or more	254 (65.5)			3.9 (0.7-21.4)	2.9 (0.4-19.7)

I. Unadj. OR: Unadjusted Odds Ratio, Adj. OR: Adjusted Odds Ratio, CI: Confidence Interval

II. *P*-value: *p < 0.01, ‡*p *< 0.00. *P*-value < 0.05 was statistically significant.

III. †Pregnant women who had a positive attitude to routine antenatal HIV testing were 98.5%. Hence there were too few cases in some cells giving rise to the wide confidence intervals of the odds ratios and inability to calculate the odds ratio for occupation.

IV. The goodness-of-fit test (Omnibus Tests of Model Coefficients) of the final model for male partner permission to test for HIV was significant [Chi-square statistic (χ^2^) = 41.434, degrees of freedom (df) = 8, *p *= 0.000] and the Hosmer and Lemeshow goodness-of-fit test was not significant [χ^2 ^= 5.563, df = 8, *p *= 0.696] as indicators of model appropriateness.

V. The goodness-of-fit test (Omnibus Tests of Model Coefficients) of the final model for HIV testing was significant [χ^2 ^= 11.025, df = 8, *p *= 0.000] and the Hosmer and Lemeshow goodness-of-fit test was not significant [χ^2 ^= 5.637, df = 8, *p *= 0.688] as indicators of model appropriateness.

### Participants' knowledge about PMTCT and infant feeding options

More than 60% of the participants knew that HIV could be passed from an infected mother to her child during pregnancy; however, about 85% of the respondents knew that mother to child transmission could occur during labour and about 89% knew it could occur through breastfeeding (Table 5). However, only 38% (147/388) of women knew the correct number of children who were likely to be infected with HIV through breastfeeding out of 10 HIV-infected women. The majority of the new antenatal attendees (89%, 347/388) knew that a pregnant woman could do something to reduce the risk of mother to child transmission of HIV during pregnancy, and 86% (335/388) of mothers knew that an HIV-infected mother could take some measures to reduce the risk of infecting her child through breastfeeding.

**Table 5:** Participants' knowledge about mother-to-child transmission of HIV and infant feeding options (N = 388), Mbale, Uganda

Questions to participants	Correct answer	Correct responses n (%)
*(1) Is it possible that when the mother or the father is HIV positive*		
*and their newborn child can be HIV negative?*	Yes	296 (76.3)
		
(2) *When can HIV be passed from a mother to her child?*		
- during pregnancy	Yes	239 (61.6)
- during labour	Yes	328 (84.5)
- through breastfeeding	Yes	344 (88.7)
- other^**a**^	Yes	129 (33.2)
		
*(3) If there are 10 HIV infected pregnant women, how many do*		
*you think would have babies born with HIV virus?(between 0-10)*	1-4	226 (58.2)
		
*(4) How many babies could get HIV infected through breastfeeding*		
*out of 10 HIV infected mothers? ....(between 0-10)*	1-3	147 (37.9)
		
*(5) What can a mother do to reduce the risk of transmission of HIV*		
*to her child during pregnancy?*		
- taking antiretroviral drugs	Yes	323 (83.2)
- having protected sex with her partner (condom use)	Yes	177 (45.6)
- other ways^**b**^	Yes	83 (21.4)
		
*(6)Can an HIV infected mother do anything to reduce the risk of*		
*transmission of HIV to her child during breastfeeding period?*	Yes	335 (86.3)
		
*(7) What can an HIV positive mother do to reduce the risk of getting*		
*her baby infected with HIV during the breastfeeding period?*		
- exclusively breastfeed for 6 months	Yes	244 (62.9)
- not breastfeeding, give infant formula	Yes	240 (61.9)
- not breastfeeding, give diluted cow's milk	Yes	268 (69.1)
- good breast care (no sore or cracked nipples)	Yes	139 (35.8)
- other ways^**c**^	Yes	76 (19.6)
		
(8) If you were HIV positive, which infant feeding option would be		
feasible to you? (Give only one answer)		
(a) - infant formula, no breast milk		45 (11.6)
(b) - cow's milk, no breast milk		233 (60.1)
(c) - breast milk only for 6 months		94 (24.2)
(d) - other^**d**^		13 (3.4)

- ^**a**^sharing sharp instruments like needles and injection needles with the baby

- ^**b**^abstaining from sexual intercourse, being faithful to your partner

- ^**c**^using drugs to prevent HIV through breast milk

- ^**d**^breastfeeding for 3 months, then giving either cow's milk or porridge (from soya/millet flour)

Out of 388 participants, 323 (83%) knew that taking antiretroviral drugs if HIV infected reduced the risk of vertical transmission of HIV during pregnancy. However, few mothers (46%, 177/388) knew that having protected sex with their partners (condom use) reduced the risk of mother to child transmission of HIV during pregnancy (Table 5). Many of the participants (63%, 244/388) knew that in order to reduce risk of vertical transmission of HIV during the breastfeeding period, an HIV-infected mother could use the infant feeding option of exclusive breastfeeding for six months. Similarly, more than 60% of respondents knew that by avoiding breastfeeding and using either infant formula or diluted cow's milk instead, an HIV-infected mother would prevent transmission of HIV to her baby through breastfeeding (Table 5).

The predictors of having knowledge of exclusive breastfeeding as one of the measures for prevention of mother to child transmission of HIV were: having completed secondary school (OR: 2.5, CI: 1.3-4.9): belonging to a non-Bagisu ethnic group (OR: 1.7, CI: 1.0-2.7); and having three or more pregnancies (OR: 2.5, CI: 1.4-4.5) (Table 1). However, only 24% (94/388) reported that they would opt for exclusive breastfeeding for six months as an infant feeding option if they were HIV infected. Instead, 60% (233/388) of the participants said they would hypothetically choose the option of using diluted cow's milk and no breast milk (Table 5).

### Study participants' suggestions for service improvement in the antenatal clinic

Although many (79%, 308/388) of the antenatal attendees rated their first visits to the antenatal clinic highly (good or very good), some of them made some suggestions for service improvement at the clinic (Additional file 1).

## Discussion

Overall, our study revealed that most of the study participants had a positive attitude towards routine antenatal HIV counselling and testing (RCT). This finding is similar to that reported in a study in Botswana [28], where 81% of participants reported that they were either extremely or very much in favour of routine testing. The high level of positive attitudes to RCT in our study could be attributed to several factors. It is possible that the pregnant women were less fearful of accepting HIV testing because this approach was offered as part of the "standard of care" given to all women in the antenatal clinic. However, a study done in six health facilities (five health centres and one hospital) in Dodoma, Tanzania, showed that about a quarter of the women were not satisfied with the counselling they received about prevention of mother to child transmission of HIV (24.8%), privacy (24%) or the waiting time spent in the clinic as they accessed the PMTCT services (28%) [29].

The majority of the new antenatal attendees rated pre-test and post-test HIV counselling highly, tested for HIV and received same-day results. Similar findings were documented in a study in urban Zimbabwe, where 100% and 99.8% of the women received pre-test and post-test HIV counselling, respectively, and 99.9% accepted routine HIV testing [12]. Similar findings were reported from studies in rural areas of Zimbabwe [30,31] and Lilongwe, Malawi [32]. The availability of rapid HIV testing in the clinic and the giving of same-day HIV test results may have contributed to the high participation in the HIV testing. However, in our study, four pregnant women tested for HIV but reported that they did not receive the test results. It is possible that they actually received their results but reported to the contrary, thinking that they were being asked to reveal their HIV sero-status. Use of rapid HIV screening tests in the antenatal clinic ensures same-day results for all mothers who accept HIV testing.

The study also found that the new antenatal attendees aged 15 to 24 years were more likely to have positive attitudes to pre-test HIV counselling. In a study done in Zambia, it was noted that readiness to test for HIV was higher among the young than among older people [33]. The positive attitude to pre-test HIV counselling among pregnant women who were either residing in urban areas or were least poor could be explained by the fact they have more access to information about HIV counselling through the print and electronic media. Therefore, they are more likely to be aware of the benefits of HIV counselling and testing.

Our finding that women who had three or more pregnancies had positive attitudes towards pre-test counselling could be explained by their previous interactions with the healthcare system, which exposed them to information on HIV testing and associated benefits. The educated women were more likely to seek permission from their male partners to test for HIV than the less educated. This is probably due to the fact that the educated women are more likely to discuss issues concerning their sexuality and health with their spouses. Those who were either married or cohabiting were almost six times more likely to ask for permission from the partners. This could be linked to the desire to obtain support from their partners, including money for transport to such health facilities. Our earlier study in the same setting revealed that the majority of the men (97%) provided financial support to their wives to access antenatal care [34].

Our study also showed that many of the antenatal attendees had correct knowledge about mother to child transmission (MTCT) of HIV and how to prevent it. Women who had completed secondary school education were more likely to have correct knowledge of exclusive breastfeeding as a preventive measure for vertical transmission of HIV. A similar finding was reported by the Botswana study [28]. Our study has revealed that pregnant women who had completed secondary education were approximately three times more likely to have good knowledge about exclusive breastfeeding. The educated have better access to health information. An earlier study done in the same region highlighted the positive influence of higher education on infant feeding practices [35].

Our study also revealed that women who had three or more pregnancies were three times more likely to have good knowledge about exclusive breastfeeding. This could be explained by their previous interaction with the healthcare system, which exposed them to information on exclusive breastfeeding and its associated benefits Many of study participants (63%) reported that exclusive breastfeeding (EBF) for six months reduced the risk of MTCT. However, few (24%) of them thought it a feasible infant feeding option if they were HIV positive; instead, many (60%) reported that they would use cow's milk.

At the time the study was conducted, modified cow's milk was one of the replacement feeding options for infants of HIV-infected mothers, according to the national policy guidelines[18], if affordable, feasible, acceptable, sustainable and safe (AFASS). However, according to the most recent WHO recommendations [36], home-modified animal milk is not recommended as a replacement food for infants in the first six months of life. In a region where breastfeeding is almost universal [37], counselling about EBF in the antenatal clinic should be intensified as studies in sub-Saharan Africa have revealed that EBF reduces postnatal HIV transmission [38-40]. Knowledge is an important determinant for behavioural change. Hence, good quality HIV counselling is important for the success of PMTCT efforts.

The study also identified some challenges to the implementation of antenatal routine HIV testing. Although the majority of the women were satisfied with the services in the antenatal clinic, some gaps were identified. These included the following: inadequate supply of drugs and equipment; shortage of midwives and/or counsellors and low male involvement in routine antenatal HIV testing services. Some women felt that individual counselling was inadequate while others felt they were pressured to test for HIV. Similar challenges have been reported from other studies in east Africa [29,41,42].

The factors hindering male involvement in the PMTCT programme have been reported in a previous study in this region [34]. As shown in this study, about 54% of the women sought permission from their spouses to have an HIV test. However, some studies have documented that some women refuse to test for HIV because of the need to seek their partners' assent [43,44]. There is need for more male involvement in antenatal HIV counselling and testing as this has been shown to increase the use of PMTCT interventions in resource-limited settings [45-47].

In a recent study in Uganda by Wabwire-Mangen and his colleagues, many (43%) of the new HIV infections in adults (15-49 years) occurred among people in discordant monogamous relationships [4]. Hence, there is a need for increased couple counselling and testing in the PMTCT programme, as recommended in the Uganda national policy on HIV counselling and testing [23]. This would most likely facilitate couples' ability to follow through on intentions and decisions made during the HIV counselling and testing sessions [48]. One way of promoting men's participation in antenatal HIV counselling and testing could be by health staff sending written notes inviting them to come to the clinic, as suggested by participants. This suggestion had been alluded to in a previous study in this study population [34].

Our study had some potential limitations. Being a cross-sectional survey, causality cannot be inferred from our findings. Although the study participants were from both rural and urban areas, they may not be representative of the whole population of Uganda. Therefore, country-wide generalization of our study findings is not implicit and it is not possible to generalize our findings to other sub-Saharan Africa countries. Since we enrolled the antenatal attendees consecutively, our study may have suffered from selection bias, thus affecting the internal validity of the study. In addition, participants' self-reports could have introduced misclassification and bias. We attempted to reduce social desirability bias by presenting study aims to the respondents in general terms. In our study, we deliberately did not ask the women about their HIV status in order to assure confidentiality and also maximize validity.

## Conclusions

Our study findings have demonstrated that antenatal routine HIV counselling and testing seems to be largely acceptable to the pregnant women in eastern Uganda and has enabled most of them to know their HIV status as part of the PMTCT package of services. To ensure good quality service in the antenatal clinic, there is a need for adequate supplies of drugs, sundries, HIV test kits and equipment, and enough numbers of health workers equipped with good counselling skills. More concerted efforts by programme managers are needed to scale up this service to antenatal clinics in lower level health units in order to maximize its potential benefits for the population. Finally, further work through research and innovative interventions is needed in order to improve male partner involvement in HIV testing in antenatal clinics.

## Competing interests

The authors declare that they have no competing interests.

## Authors' contributions

RB participated in the conception, design and implementation of the study, statistical analysis, interpretation and drafting of the manuscript. JKT participated in the design, and implementation of the study, interpretation and drafting of the manuscript. GN participated in the design of the study, interpretation and drafting of the manuscript. CASK participated in interpretation and the drafting on the manuscript. TT participated in the conception and design of the study, interpretation and drafting the manuscript. All authors read and approved the final manuscript.

## Supplementary Material

Additional file 1 **Study participants’ suggestions about service improvement in antenatal clinic in Mbale Regional Referral Hospital, Uganda**. Click here for file

## References

[B1] WHO Guidance on provider-initiated HIV testing and counselling in health facilities 2007 Geneva, Switzerland http://www.who.int/mediacentre/news/releases/2007/pr24/en/index.html

[B2] MOH/ORC Uganda HIV/AIDS Sero-behavioural Survey 2004-2005 2006 Calverton, Maryland, USA: Ministry of Health [Uganda] and ORC Macro [USA] 194 http://www.measuredhs.com/pubs/pdf/AIS2/AIS2.pdf

[B3] UNAIDS/WHO AIDS epidemic update 2009 Geneva 100 http://www.who.int/hiv/pub/epidemiology/epidemic/en/index.html

[B4] Wabwire-Mangen F Odiit M Kirungi W Mwijuka B Kaweesa DK Wanyama J M H Uganda: HIV modes of transmission and prevention response analysis 2009 Kampala: Uganda AIDS Commission, Republic of Uganda and UNAIDS

[B5] CDC Revised Recommendations for HIV Screening of Pregnant Women MMWR Recomm Rep 2001 13 Atlanta: CDC 59 86 http://www.cdc.gov/mmwr/preview/mmwrhtml/rr5019a2.htm 11718473

[B6] CDC HIV Testing Among Pregnant Women - United States and Canada, 1998-2001 MMWR 2002 13 Atlanta: CDC 1013 1016 http://www.cdc.gov/mmwr/preview/mmwrhtml/mm5145a1.htm 12458916

[B7] Jayaraman GC Preiksaitis JK Larke B Mandatory reporting of HIV infection and opt-out prenatal screening for HIV infection: effect on testing rates CMAJ 2003 13 6 679 682 12642422 PMC154912

[B8] Simpson WM Johnstone FD Goldberg DJ Gormley SM Hart GJ Antenatal HIV testing: assessment of a routine voluntary approach BMJ 1999 13 7199 1660 1661 10373168 10.1136/bmj.318.7199.1660 PMC28144

[B9] Stringer EM Stringer JSA Cliver SP Goldenberg RL Goepfert AR Evaluation of a new testing policy for human immunodeficiency virus to improve screening rates Obstetrics & Gynecology 2001 13 6 1104 1108 10.1016/S0029-7844(01)01631-3 11755561

[B10] Sherr L Fox Z Lipton M Whyte P Jones P Harrison U Group CIS Sustaining HIV testing in pregnancy - Evaluation of routine offer of HIV testing in three London hospitals over 2 years AIDS Care: Psychological and Socio-medical Aspects of AIDS/HIV 2006 13 3 183 188 10.1080/09540120500456599 16615201

[B11] Creek TL Ntumy R Seipone K Smith M Mogodi M Smit M Legwaila K Molokwane I Tebele G Mazhani L Shaffer N Kilmarx PH Successful Introduction of Routine Opt-Out HIV Testing in Antenatal Care in Botswana Journal of Acquired Immune Deficiency Syndromes 2007 13 1 102 107 10.1097/QAI.0b013e318047df88 17460473

[B12] Chandisarewa W Stranix-Chibanda L Chirapa E Miller A Simoyi M Mahomva A Maldonado Y Shetty AK Routine offer of antenatal HIV testing ("opt-out" approach) to prevent mother-to-child transmission of HIV in urban Zimbabwe Bulletin of the World Health Organization 2007 13 843 850 18038074 10.2471/BLT.06.035188 PMC2636259

[B13] Kasenga F Byass P Hurtig AK Emmelin M The implications of policy changes on the uptake of a PMTCT programme in rural Malawi: first three years of experience Global Health Action 2009 13 *incl Supplements*. 10.3402/gha.v2i0.1883 PMC2779935 20027274

[B14] CDC Introduction of routine HIV testing in prenatal care--Botswana, 2004 MMWR Morb Mortal Wkly Rep 2004 13 Atlanta, USA: CDC 1083 1086 http://www.cdc.gov/mmwr/preview/mmwrhtml/mm5346a2.htm 15565017

[B15] Bassett IV Giddy J Nkera J Wang B Losina E Lu Z Freedberg KA Walensky RP Routine Voluntary HIV Testing in Durban, South Africa: The Experience From an Outpatient Department Journal of Acquired Immune Deficiency Syndromes 2007 13 2 181 186 10.1097/QAI.0b013e31814277c8 17667332 PMC2140230

[B16] UNAIDS/WHO UNAIDS/WHO policy statement on HIV testing 2004 Geneva http://www.who.int/hiv/pub/vct/statement/en/index.html

[B17] CDC Revised Recommendations for HIV Testing of Adults, Adolescents, and Pregnant Women in Health-Care Settings Morbidity and Mortality Weekly Report (MMWR), Recommendations and reports 2006 13 Atlanta: Centers for Disease Control and Prevention (CDC) 1 17 http://www.cdc.gov/mmwr/preview/mmwrhtml/rr5514a1.htm 16988643

[B18] Ministry of Health (MOH) Uganda Policy guidelines for prevention of mother-to-child transmission of HIV 2006 Kampala

[B19] Byamugisha R Tylleskar T Kagawa M Onyango S Karamagi C Tumwine J Dramatic and sustained increase in HIV-testing rates among antenatal attendees in Eastern Uganda after a policy change from voluntary counselling and testing to routine counselling and testing for HIV: a retrospective analysis of hospital records, 2002-2009 BMC Health Services Research 2010 13 1 290 10.1186/1472-6963-10-290 20946632 PMC2964695

[B20] Uganda Bureau of Statistics (UBOS) Recent releases (2010 Statistical Abstract Released) 2010 Kampala

[B21] Uganda Bureau of Statistics 2002 Uganda Population and Housing Census, Main Report (released in March 2005) 2005 Kampala http://www.ubos.org/index.php?st=pagerelations2&id=16&p=related pages 2:2002Census Results

[B22] STD/AIDS Control Programme. Ministry of Health Uganda STD/HIV/AIDS Surveillance Report, June 2003 2003 Kampala

[B23] Ministry of Health (MOH) Uganda Uganda National Policy on HIV Counselling and Testing 2005 Kampala

[B24] Vyas S Kumaranayake L Constructing socio-economic status indices: how to use principal components analysis Health Policy Plan 2006 13 6 459 468 10.1093/heapol/czl029 17030551

[B25] Tabachnick B Fidell L Using multivariate statistics 1996 3 New York, NY HarperCollins College Publishers

[B26] Filmer D Pritchett LH Estimating Wealth Effects without Expenditure Data-or Tears: An Application to Educational Enrollments in States of India Demography 2001 13 1 115 132 11227840 10.1353/dem.2001.0003

[B27] Howe L Hargreaves J Huttly S Issues in the construction of wealth indices for the measurement of socio-economic position in low-income countries Emerging Themes in Epidemiology 2008 13 1 3 10.1186/1742-7622-5-3 18234082 PMC2248177

[B28] Weiser SD Heisler M Leiter K Percy-de Korte F Tlou S DeMonner S Phaladze N Bangsberg DR Iacopino V Routine HIV Testing in Botswana: A Population-Based Study on Attitudes, Practices, and Human Rights Concerns PLoS Med 2006 13 7 e261 10.1371/journal.pmed.0030261 16834458 PMC1502152

[B29] Lyatuu MB Msamanga G Kalinga AK Client's satisfaction with services for prevention of mother to child transmission of HIV in Dodoma rural district East Afr J Public Health 2008 13 3 174 179 19374320 10.4314/eajph.v5i3.38999

[B30] Perez F Zvandaziva C Engelsmann B Dabis F Acceptability of Routine HIV Testing ("Opt-Out") in Antenatal Services in Two Rural Districts of Zimbabwe Journal of Acquired Immune Deficiency Syndromes 2006 13 4 514 520 10.1097/01.qai.0000191285.70331.a0 16652062

[B31] Mugore L Engelsmann B Ndoro T Dabis F Perez F An assessment of the understanding of the offer of routine HIV testing among pregnant women in rural Zimbabwe AIDS Care: Psychological and Socio-medical Aspects of AIDS/HIV 2008 13 6 660 666 10.1080/09540120701687034 18576167

[B32] Moses A Zimba C Kamanga E Nkhoma J Maida A Martinson F Mofolo I Joaki G Muita J Spensley A Hoffman I van der Horst C Prevention of mother-to-child transmission: program changes and the effect on uptake of the HIVNET 012 regimen in Malawi AIDS 2008 13 1 83 87 10.1097/QAD.0b013e3282f163b5 18090395

[B33] Fylkesnes K Siziya S A randomized trial on acceptability of voluntary HIV counselling and testing Tropical Medicine & International Health 2004 13 5 566 572 10.1111/j.1365-3156.2004.01231.x 15117300

[B34] Byamugisha R Tumwine J Semiyaga N Tylleskar T Determinants of male involvement in the prevention of mother-to-child transmission of HIV programme in Eastern Uganda: a cross-sectional survey Reproductive Health 2010 13 1 12 10.1186/1742-4755-7-12 20573250 PMC2913932

[B35] Fadnes L Engebretsen I Wamani H Semiyaga N Tylleskar T Tumwine J Infant feeding among HIV-positive mothers and the general population mothers: comparison of two cross-sectional surveys in Eastern Uganda BMC Public Health 2009 13 1 124 10.1186/1471-2458-9-124 19422709 PMC2687447

[B36] WHO HIV and infant feeding: Revised Principles and Recommendations [Rapid Advice 2009 Geneva http://www.who.int/hiv/pub/paediatric/advice/en/index.html

[B37] Nankunda J Tylleskär T Ndeezi G Semiyaga N Tumwine JK Establishing individual peer counselling for exclusive breastfeeding in Uganda: implications for scaling-up Maternal & Child Nutrition 2010 13 1 53 66 10.1111/j.1740-8709.2009.00187.x 20055930 PMC6860637

[B38] Coovadia HM Rollins NC Bland RM Little K Coutsoudis A Bennish ML Newell ML Mother-to-child transmission of HIV-1 infection during exclusive breastfeeding in the first 6 months of life: an intervention cohort study The Lancet 2007 13 9567 1107 1116 10.1016/S0140-6736(07)60283-9 17398310

[B39] Kuhn L Sinkala M Kankasa C Semrau K Kasonde P Scott N Mwiya M Vwalika C Walter J Tsai WY Aldrovandi GM Thea DM High Uptake of Exclusive Breastfeeding and Reduced Early Post-Natal HIV Transmission PLoS ONE 2007 13 12 e1363 10.1371/journal.pone.0001363 18159246 PMC2137948

[B40] Piwoz EG Humphrey JH Tavengwa NV Iliff PJ Marinda ET Zunguza CD Nathoo KJ Mutasa K Moulton LH Ward BJ The Impact of Safer Breastfeeding Practices on Postnatal HIV-1 Transmission in Zimbabwe Am J Public Health 2007 13 7 1249 1254 10.2105/AJPH.2006.085704 17538064 PMC1913077

[B41] Evans C Ndirangu E The nursing implications of routine provider-initiated HIV testing and counselling in sub-Saharan Africa: A critical review of new policy guidance from WHO/UNAIDS International Journal of Nursing Studies 2009 13 5 723 731 10.1016/j.ijnurstu.2008.11.003 19159879

[B42] Medley AM Kennedy CE Provider Challenges in Implementing Antenatal Provider-Initiated HIV Testing and Counseling Programs in Uganda AIDS Education and Prevention 2010 13 2 87 99 10.1521/aeap.2010.22.2.87 20387980

[B43] Homsy J King R Malamba SS Opio C Kalamya JN Mermin J Okallanyi A Obonyo JH The Need for Partner Consent Is a Main Reason for Opting Out of Routine HIV Testing for Prevention of Mother-to-Child Transmission in a Rural Ugandan Hospital Journal of Acquired Immune Deficiency Syndromes 2007 13 3 366 369 17327761 10.1097/QAI.0b013e31802f1303

[B44] Semrau K Kuhn L Vwalika C Kasonde P Sinkala M Kankasa C Shutes E Aldrovandi G Thea DM Women in couples antenatal HIV counseling and testing are not more likely to report adverse social events AIDS 2005 13 6 603 609 10.1097/01.aids.0000163937.07026.a0 15802979 PMC1201374

[B45] Farquhar C Kiarie JN Richardson BA Kabura MN John FN Nduati RW Mbori-Ngacha DA John-Stewart GC Antenatal couple counseling increases uptake of interventions to prevent HIV-1 transmission J Acquir Immune Defic Syndr 2004 13 5 1620 1626 10.1097/00126334-200412150-00016 15577420 PMC3384734

[B46] Karl P Motlatso M Nancy PM Rendani L Determinants of adherence to a single-dose nevirapine regimen for the prevention of mother-to-child HIV transmission in Gert Sibande district in South Africa Acta Pædiatrica 2010 13 5 699 704 10.1111/j.1651-2227.2010.01699.x 20146724

[B47] Kiarie JN Kreiss JK Richardson BA John-Stewart GC Compliance with antiretroviral regimens to prevent perinatal HIV-1 transmission in Kenya AIDS 2003 13 1 65 71 10.1097/00002030-200301030-00009 12478070 PMC3387271

[B48] Painter TM Voluntary counseling and testing for couples: a high-leverage intervention for HIV/AIDS prevention in sub-Saharan Africa Social Science & Medicine 2001 13 11 1397 1411 10.1016/S0277-9536(00)00427-5 11710416

