# Supplementary material for: Attitudes to routine HIV counselling and testing, and knowledge about prevention of mother to child transmission of HIV in eastern Uganda: a cross-sectional survey among antenatal attendees
Source: J Int AIDS Soc. 2010 Dec 13;13:52. doi: 10.1186/1758-2652-13-52 (PMC3017012; doi:10.1186/1758-2652-13-52)
Supplement: Additional file 1 — Study participants’ suggestions about service improvement in antenatal clinic in Mbale Regional Referral Hospital, Uganda. [file 1758-2652-13-52-S1.DOC]

# Additional files

## Additional file 1. Study participants’ suggestions about service improvement in antenatal clinic in Mbale Regional Referral Hospital, Uganda

| **ANC generally** |
| --- |
| - There is need for improved customer care skills and teamwork among the clinic staff |
| - Increase number of midwives in order to reduce waiting time for mothers in the clinic |
| - Have adequate stocks of drugs like haematinics (iron and folate), anti-malarial and de-worming agents |
| - Have enough equipment like weighing scales, tape measures and sphygmomanometers (machines for measuring blood pressure) |
| - Need to expand the clinic space and provide enough seats to accommodate all the mothers |
| - ANC attendees should be given a note to take to their husbands to escort them for ANC visits and HIV testing |
| **PMTCT specifically** |
| - Increase use of the TV and films when giving health education to ease understanding of the health messages |
| - Need to ensure verbal consent from the mothers before routine HIV testing |
| - Need for adequate individual HIV counselling |
| - HIV-positive mothers be given ongoing support and education about infant feeding options |
| - Should avoid stock outs of the PMTCT drugs in the clinic |
| - All units in the hospital should have HIV counselling and testing services to avoid missed opportunities |
